# Unilateral diaphragm paralysis: a dysfunction restricted not just to one hemidiaphragm

**DOI:** 10.1186/s12890-018-0698-1

**Published:** 2018-08-02

**Authors:** Mayra Caleffi-Pereira, Renata Pletsch-Assunção, Letícia Zumpano Cardenas, Pauliane Vieira Santana, Jeferson George Ferreira, Vinícius Carlos Iamonti, Pedro Caruso, Angelo Fernandez, Carlos Roberto Ribeiro de Carvalho, André Luís Pereira Albuquerque

**Affiliations:** 1 Pulmonary Division, Heart Institute (Incor), Hospital das Clínicas da Faculdade de Medicina da Universidade de São Paulo, Av. Dr. Enéas Carvalho Aguiar, n°44. Cerqueira Cesar., São Paulo, 05403-900 Brazil; 2 Thoracic Surgery Division, Heart Institute (Incor), Hospital das Clínicas da Faculdade de Medicina da Universidade de São Paulo, São Paulo, Brazil; 3 Sírio-Libanês Teaching and Research Institute, São Paulo, Brazil

**Keywords:** Diaphragm paralysis, Diaphragm strength, Diaphragm dysfunction, Electromagnetic phrenic nerve stimulation

## Abstract

**Background:**

Most patients with unilateral diaphragm paralysis (UDP) have unexplained dyspnea, exercise limitations, and reduction in inspiratory muscle capacity. We aimed to evaluate the generation of pressure in each hemidiaphragm separately and its contribution to overall inspiratory strength.

**Methods:**

Twenty-seven patients, 9 in right paralysis group (RP) and 18 in left paralysis group (LP), with forced vital capacity (FVC) < 80% pred, and 20 healthy controls (CG), with forced expiratory volume in 1 s (FEV_1_) > 80% pred and FVC > 80% pred, were evaluated for lung function, maximal inspiratory (MIP) and expiratory (MEP) pressure measurements, diaphragm ultrasound, and transdiaphragmatic pressure during magnetic phrenic nerve stimulation (Pdi_Tw_).

**Results:**

RP and LP had significant inspiratory muscle weakness compared to controls, detected by MIP (− 57.4 ± 16.9 for RP; − 67.1 ± 28.5 for LP and − 103.1 ± 30.4 cmH_2_O for CG) and also by Pdi_TW_ (5.7 ± 4 for RP; 4.8 ± 2.3 for LP and 15.3 ± 5.7 cmH_2_O for CG). The Pdi_Tw_ was reduced even when the non-paralyzed hemidiaphragm was stimulated, mainly due to the low contribution of gastric pressure (around 30%), regardless of whether the paralysis was in the right or left hemidiaphragm. On the other hand, in CG, esophagic and gastric pressures had similar contribution to the overall Pdi (around 50%). Comparing both paralyzed and non-paralyzed hemidiaphragms, the mobility during quiet and deep breathing, and thickness at functional residual capacity (FRC) and total lung capacity (TLC), were significantly reduced in paralyzed hemidiaphragm. In addition, thickness fraction was extremely diminished when contrasted with the non-paralyzed hemidiaphragm.

**Conclusions:**

In symptomatic patients with UDP, global inspiratory strength is reduced not only due to weakness in the paralyzed hemidiaphragm but also to impairment in the pressure generated by the non-paralyzed hemidiaphragm.

## Background

Dysfunction of the diaphragm is an important, underdiagnosed cause of breathlessness. Most patients with bilateral involvement already experience dyspnea on mild effort or even at rest, mainly while in the supine position. In unilateral diaphragm weakness, the presentation can be asymptomatic or with dyspnea on exercise, frequently discovered incidentally [1, 2]. The association of comorbidities, such as cardiopulmonary diseases or obesity, frequently increases the clinical symptoms, but the mechanisms involved in the genesis of dyspnea still have not been elucidated regarding unilateral involvement.

During normal inspiration, the contraction of the diaphragm draws a caudal movement as a whole. In other words, both right and left sides work harmoniously generating positive pressure in the abdominal compartment and negative pressure in the pleural space. In unilateral diaphragm paralysis (UDP), it is believed that the non-paralyzed hemidiaphragm increases in strength to compensate for the dysfunction of the paralyzed hemidiaphragm; therefore, most of these patients are able to maintain appropriate ventilatory conditions at rest and during mild exercise [3]. However, this is not true for all patients, because many of them have unexplained dyspnea, exercise limitations, and a reduction in inspiratory muscle capacity [1, 4]. Taking into account the fact that the paralyzed hemidiaphragm usually has a pendulum movement into the thorax (paradoxical movement) [1, 2], it has the potential to impair the pressure generated during inspiration even in the non-paralyzed hemidiaphragm.

We hypothesized that UDP also impairs the function of the non-paralyzed hemidiaphragm, mainly influenced by the low abdominal pressure. The aim of the present study was to evaluate the generation of pressure in each hemidiaphragm separately and their contribution to the overall transdiaphragmatic pressure (Pdi) in healthy subjects and patients with UDP.

## Methods

### Subjects

This was a cross-sectional study involving 27 patients with UDP (9 with right paralysis [RP] and 18 with left paralysis [LP]), who were consecutively recruited from a tertiary university hospital. All diagnoses were confirmed by the respiratory physician, using complementary imaging studies with ultrasound (USG), chest computed tomography, or radiography. The inclusion criteria were the restrictive pattern (FVC < 80% pred) and body mass index (BMI) between 20 and 30 kg/m^2^. Patients with lung diseases and neuromuscular disorders were excluded. A control group (CG) was included composed of 20 healthy, age-matched subjects with normal lung function (FEV_1_ > 80% pred and FVC > 80% pred) who were physically inactive (exercise activity less than twice a week). The study was approved by the local Ethics Committee (CapPesq) (protocol number: 0835/11), and all subjects provided written informed consent.

### Study protocol

The individuals were evaluated with the following measurements at rest: lung function tests, maximal inspiratory (MIP) and expiratory (MEP) pressures, esophageal and gastric pressures during electromagnetic phrenic nerve stimulation with Twitches, and diaphragm motion and thickness with ultrasound. All the measurements were completed during a single visit.

### Measurements

#### Lung function tests

All measurements were performed according to American Thoracic Society/European Respiratory Society (ATS/ERS) guidelines [5–7]. Spirometry was performed using a calibrated pneumotachograph (Medical Graphics Corporation - MGC, St. Paul, MN, USA), whereas lung volumes and carbon monoxide diffusion capacity (DL_CO_) were obtained on a body plethysmograph (Elite Dx, Elite Series™ – MGC). The following variables were obtained: forced vital capacity (FVC), expiratory forced volume in the first second (FEV_1_), total lung capacity (TLC), residual volume (RV), and DL_CO_. The control group underwent only forced spirometry to measure FEV_1_ and FVC.

#### Dyspnea

The degree of dyspnea was assessed using the Medical Research Council Breathlessness Scale [8].

#### Maximal inspiratory (MIP) and expiratory (MEP) pressures

MIP and MEP were measured using a digital manovacuometer (MicroRPM®, CareFusion, Yorba Linda, CA, USA). In the sitting position with a nose clip, subjects were asked to exhale to RV and then perform a maximal inspiratory effort for at least 3 s for MIP measurement; for MEP measurement, they were asked to inhale to TLC followed by a maximal expiratory effort for at least 3 s. The maneuvers were repeated 3 to 5 times, and the highest pressures were selected for analysis [9].

The predicted values for lung function and ventilatory pressures were derived from the Brazilian population [10–12].

#### Transdiaphragmatic pressure (Pdi)

To measure Pdi, 2 air-filled balloon catheters (Adult Esophageal Balloon Catheter Set, CooperSurgical, Trumbull, CT, USA) were used. One was positioned into the distal esophagus and filled with 1 mL of air, ensuring the correct position by correlating esophageal pressure (Pes) with mouth pressure (Pmo) by using the occlusion technique [13]. To access gastric pressure (Pga), the distal balloon was introduced 65 cm from the nares and positioned in the stomach, filled with 1.5 mL of air. Both catheters were connected to pressure transducers (TruStability® Standard Accuracy Silicon Ceramic, Honeywell, Morris Plains, NJ, USA), which were calibrated before each test with a graded water column at 3 different levels (0, 10, and 20 cmH_2_O). Both Pes and Pga were measured continuously, and Pdi was calculated automatically (Pdi = Pga – Pes) during the entire acquisition.

To measure non-volitional Pdi we performed magnetic phrenic nerve stimulation (Twitch) (MagProCompact®, MagVenture-Denmark), with two 45-mm figure-of-eight coils (MC-B35, MagPro, MagVenture), to create a field focused on the phrenic nerve [14]. With the patient in a sitting position, arms relaxed, with a nose clip and a mouthpiece, the coils were placed on the posterior edge of the sternocleidomastoid muscle at the level of the cricoid cartilage [15]. The individuals were instructed to exhale to the functional residual capacity (FRC), the mouthpiece was occluded, and a maximal stimulus was applied 5 times, with 30-s intervals between them. At the time of magnetic stimulation, the Pes and Pmo were mandatory to be in plateau after expiration to ensure the Twitch was applied from the FRC. This protocol was performed in 3 phrenic magnetic stimulations: bilateral (two 45-mm coil synchronized), unilateral right, and unilateral left. The highest transdiaphragmatic pressure during magnetic phrenic nerve stimulation (Pdi_Tw_) value among at least 3 reproducible maneuvers (< 10% of difference) in each position was considered for analyses.

AqDados 7.0 software (Lynx technology, Brazil) was used for data acquisition and the AqAnalysis 7.0 (Lynx technology, Brazil) for data analysis.

#### Diaphragm ultrasound

Ultrasound imaging of the diaphragm was performed using a portable ultrasound system (Nanomaxx; Sonosite, Bothell, WA, USA) while subjects were in a semi-recumbent position. For the evaluation of diaphragmatic mobility, we installed a 2–5 MHz convex transducer. For the right hemidiaphragm, the probe was placed over the right anterior subcostal region between the midclavicular and anterior axillary lines. The transducer was directed medially cephalad, and dorsally, so that the ultrasound beam reached perpendicularly the posterior third of the right hemidiaphragm. The 2-dimensional (2D) mode was initially used to visualize and obtain the best approach, with the liver serving as an acoustic window to the right, and to select the exploration line. Then, the M-mode was used to display and measure the amplitude of the cranio-caudal diaphragmatic excursion during quiet breathing and deep breathing. For the left measurement, the probe was placed on subcostal or low intercostal regions between the anterior and mid axillary lines to obtain the best image of the left hemidiaphragm dome. The same respiratory maneuvers were performed to measure the left hemidiaphragm excursion [16, 17]. The ultrasound images acquired were measured on scans off-line using ImageJ software (available at: http://rsb.info.nih.gov/ij/docs/index.html). We recorded the averaged value of 3 consecutive measurements. The mobility of the diaphragm during a sniff test was performed to exclude paradoxical movement.

Right and left diaphragm thickness was measured in B-mode with a 6–13 MHz linear transducer placed over the zone of apposition (ZA) of the diaphragm to the rib cage, between the anterior and medial axillary lines. In the ZA, the diaphragm is observed as a structure made of 3 distinct layers: a nonechogenic central layer bordered by 2 echogenic layers, the peritoneum, and the diaphragmatic pleurae. The thickness was measured from the middle of the pleural line to the middle of the peritoneal line [18, 19]. We measured the thickness of the diaphragm during quiet spontaneous breathing at FRC, and at breath holding after a maximal inspiratory effort at TLC. Again, the averaged value of 3 consecutive measurements was recorded for each. We also calculated the thickening fraction (TF, proportional thickening of the diaphragm from FRC to TLC), an index of diaphragmatic thickening as defined by the following equation: TF = ([Th_TLC_ − Th_FRC_] / Th_FRC_) × 100, where Th_FRC_ is thickness of the diaphragm measured at the end of a quiet expiration (at FRC), and Th_TLC_ is the maximum thickness of the diaphragm measured at the end of deep breathing (at TLC).

### Statistical analysis

Statistical analysis was performed using SPSS 21.0 software (IBM SPSS Statistics®, US). The Shapiro-Wilk test was used to verify the normality of data distribution. The data are presented as mean ± standard deviation and percentage. To compare anthropometric data, lung function, and respiratory strength among the 3 groups (CG, RP, and LP), we used 1-way ANOVA with the Tukey posthoc test. The *t* test for independent samples was used to compare lung capacities (TLC, RV, and DLCO) between RP and LP, and also to compare the mobility and thickness between paralyzed hemidiaphragm and non-paralyzed hemidiaphragm. The significance level was set to 5% (*p* < 0.05).

## Results

The most frequent causes of diaphragm paralysis (DP) were trauma and idiopathic reasons. Among the comorbidities, systemic arterial hypertension was prevalent in almost half of patients, with former smokers being 30% (Table 1). None of the subjects had airflow obstruction (all had FEV_1_/FVC > the lower limit of normal and RV preserved) or lung parenchyma involvement (emphysema or air trapping areas) on lung function and chest CT, respectively.

**Table 1:** Causes of diaphragmatic paralysis, associated comorbidities, and smoking history

	n (%)
Causes of DP	
Idiopathic	8 (29.6)
Trauma	10 (37.0)
Cardiac surgery	6 (22.2)
Thoracic surgery	3 (11.1)
Comorbidities	
Arterial hypertension	12 (44.4)
Valvopathy	4 (14.8)
Acute myocardial infarction	2 (7.4)
Active smoker	–
Former smoker	8 (29.6)
Pack/years	19.6

Data expressed as n (%). *DP* diaphragmatic paralysis

No difference was found related to sex, age, and BMI among the groups (Table 2). Patients with UDP had lower FEV_1_ and FVC than controls, irrespective of the hemidiaphragm involved. Furthermore, patients were characterized by reduced lung volumes (FVC and TLC), with a normal or mild reduction in DL_CO_, and irrespective of which hemidiaphragm was involved, they had dyspnea with mild and moderate exercise (MRC score) (Table 2).

**Table 2:** Anthropometric data, lung function, and dyspnea of CG, RP, and LP

	CG (*n* = 20)	RP (*n* = 9)	LP (*n* = 18)
Anthropometric data			
Male, n (%)	10 (50)	3 (33.3)	12 (66.7)
Age, years	50 ± 6.5	57 ± 11	55 ± 11.4
BMI, kg/m^2^	27.9 ± 2.1	29.4 ± 4.6	27.9 ± 3.0
Lung Function			
FEV_1_, L % predicted	2.9 ± 0.5 91.9 ± 8.5	1.5 ± 0.6* 52.9 ± 15.3*	2.0 ± 0.7* 60.9 ± 14.1*
FVC, L % predicted	3.5 ± 0.7 91.6 ± 8.1	1.9 ± 0.6* 56.0 ± 13.2*	2.6 ± 0.8* 64.4 ± 13.7*
FEV_1_/ FVC	82.2 ± 4.9	77.0 ± 9.0*	75.8 ± 6.6*
TLC, L % predicted	–	3.9 ± 0.9 76.9 ± 11.8	4.6 ± 1.0 78.8 ± 12.1
RV, L % predicted	–	2.1 ± 0.8 119.2 ± 46.7	1.9 ± 0.5 94.9 ± 20.9
DL_CO_ % predicted	–	16.2 ± 9.9 61.9 ± 27.1	23.3 ± 7.6 82.9 ± 24.3
MRC	–	2 ± 0.7	1.5 ± 0.7

Data expressed as mean ± standard deviation and n (%) for sex. *CG* control group, *RP* right paralysis group, *LP* left paralysis group, *BMI* body mass index, *FEV*_*1*_ forced expiratory volume in 1 s, *FVC* forced vital capacity, *TLC* total lung capacity, *RV* residual volume, *L* liters, *DL*_*CO*_ carbon monoxide diffusing capacity, *MRC* Medical Research Council Breathlessness Scale. *p < 0.05 compared with control

Table 3 shows muscle strength measurements. Considering inspiratory and expiratory strength measures, patients with UDP had significant respiratory muscle weakness detected by volitional tests. No statistical difference existed in transdiaphragmatic pressure during sniff (Pdi_sniff_) between RP and LP groups compared with controls.

**Table 3:** Respiratory muscle strength data of CG, RP, and LP

	CG (*n* = 20)	RP (*n* = 9)	LP (*n* = 18)
Respiratory muscle strength			
MIP, cmH_2_O % predicted	−103.1 ± 30.4 99.5 ± 20.9	−57.4 ± 16.9* 59.9 ± 13.4*	−67.1 ± 28.5* 63.4 ± 21.2*
MEP, cmH_2_O % predicted	120.9 ± 41.6 115.5 ± 35.6	78.8 ± 24.8* 85.1 ± 24.8*	95.8 ± 43.8* 85.8 ± 32.3*
SNIP, cmH_2_O % predicted	95.9 ± 20.0 88.5 ± 28.0	53.9 ± 16.7* 56.0 ± 15.2*	62.0 ± 17.5* 60.0 ± 13.7*
Pdi_Sniff_, cmH_2_O	70.3 ± 25.5	51.7 ± 10.9	53.4 ± 21.6

Data expressed as mean ± standard deviation. *CG* control group, *Pdi* transdiaphragmatic pressure, *RP* right paralysis group, *LP* left paralysis group, *MIP* maximal inspiratory pressure, *MEP* maximal expiratory pressure, *SNIP* sniff nasal inspiratory pressure. *p < 0.05 compared with control

**Fig. 1:**
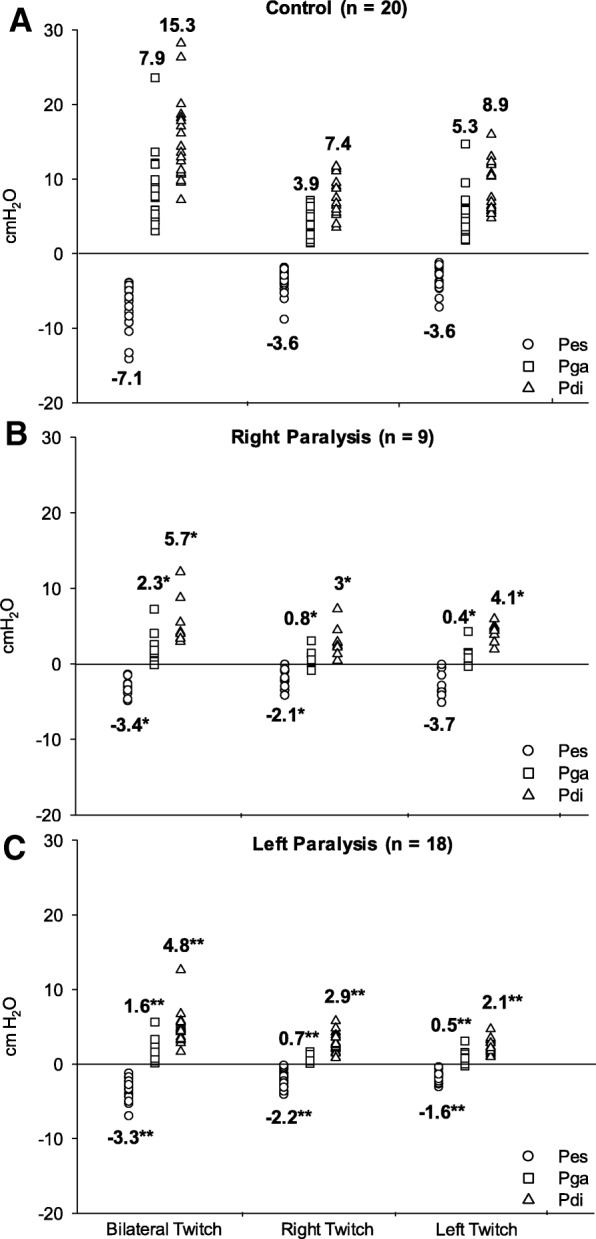
Pes, Pga, and Pdi during bilateral, right, and left Twitch. **a** Control; **b** Right paralysis; **c** Left paralysis. Pes: esophageal pressure; Pga: gastric pressure; Pdi: transdiaphragmatic pressure. *p < 0.05 Right Paralysis vs Control; **p < 0.05 Left Paralysis vs Control. Bold numbers mean the mean value (cmH_2_O)

During bilateral Twitch, the controls had Pes, Pga, and Pdi approximately 2 to 3 times higher than RP and LP. In controls, Pdi produced by unilateral stimulation was similar for both right (7.4 cmH_2_O) and left (8.9 cmH_2_O) hemidiaphragms, suggesting that each hemidiaphragm contributed equally to total Pdi during bilateral stimulation (15.3 cmH2O). In patients, unilateral Pdi was reduced not only during paralyzed-hemidiaphragm stimulation but also during non-paralyzed hemidiaphragm stimulation in both RP group (3 cmH_2_O for right twitch and 4.1 cmH_2_O for left twitch) and LP group (2.1 cmH_2_O for left twitch and 2.9 cmH_2_O for right twitch), contributing to their decreased total Pdi during bilateral stimulation (5.7 cmH_2_O for RP and 4.8 cmH_2_O for LP) compared with controls. (Fig. 1).

**Fig. 2:**
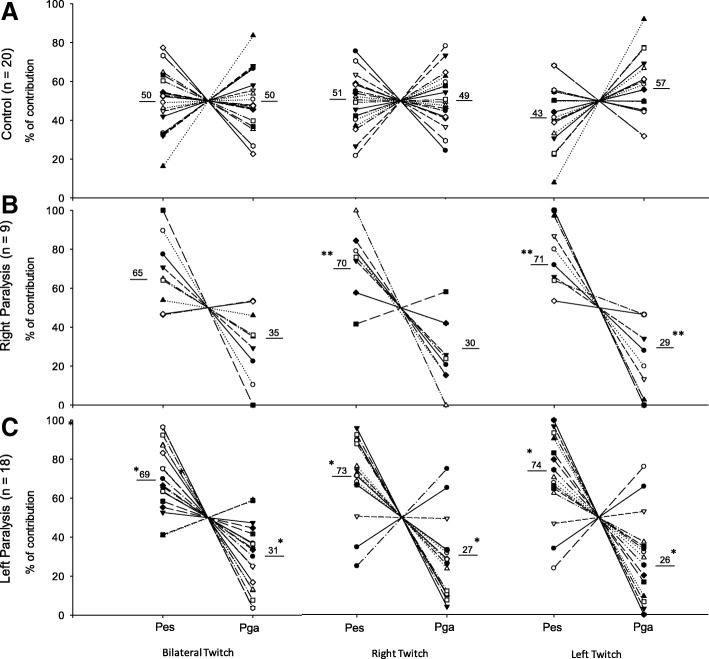
Pes and Pga contributions to Pdi during bilateral, right, and left Twitch. **a** Control; **b** Right paralysis; **c** Left paralysis. Pes: esophageal pressure; Pga: gastric pressure. **p < 0.05 Right Paralysis vs Control; *p < 0.05 Left Paralysis vs Control. Underlined numbers mean the mean value (%)

Pes and Pga have nearly equal contributions to Pdi in CG for all Twitch conditions. In patients with UDP, however, the contribution of Pga (around 30% of total Pdi) was significantly reduced compared to controls, regardless of the side of involvement (RP or LP), and the Pes was the main component for total Pdi (around 70% of total Pdi) (Fig. 2).

**Fig. 3:**
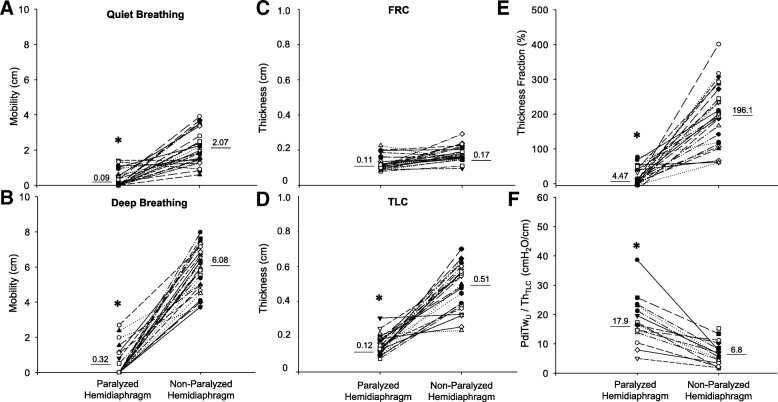
Ultrasound data of the paralyzed and normal hemidiaphragms. Mobility during quiet breathing (**a**) and deep breathing (**b**); Thickness during FRC (**c**) and TLC (**d**); Thickness Fraction (**e**) and PdiTW_U_ / Th_TLC_ ratio (**f**). FRC: forced residual capacity; TLC: total lung capacity; PdiTW_U_ / Th_TLC_: ratio between transdiaphragmatic pressure during unilateral stimuli and thickness during TLC. *p < 0.001 paralyzed hemidiaphragm vs non-paralyzed hemidiaphragm. Underlined numbers mean the mean value

The paralyzed hemidiaphragm had major impairments related to mobility and thickness compared with the non-paralyzed hemidiaphragm. The mobility, defined as total diaphragm displacement during inspiration, was significantly reduced not only during quiet breathing but also during deep breathing. The thickness was also reduced at FRC and TLC, and the thickness fraction was extremely diminished when contrasted with the non-paralyzed hemidiaphragm. Moreover, the ratio between transdiaphragmatic pressure during unilateral stimuli and thickness at TLC (PdiTw_U_/Thick_TLC_) is significantly higher in paralyzed hemidiaphragm than non-paralyzed hemidiaphragm (Fig. 3). Paradoxical movement of the paralyzed hemidiaphragm during sniff was found in 11 (38%) patients. It was not possible to evaluate sniff in 4 patients, because the left hemidiaphragm was not properly accessible in the USG window.

## Discussion

This study describes the contribution of each hemidiaphragm to the inspiratory pressure generated in patients with UDP during Twitch and compared with healthy individuals. Of note, the reduced inspiratory pressure is not restricted to the side with paralysis. Regardless of whether it is right or left paralysis, the maximal inspiratory pressures generated by paralyzed and non-paralyzed hemidiaphragms were lower, with a significantly diminished unilateral Pdi_TW_ compared with healthy controls. The drop in inspiratory pressure was mainly attributed to a significant reduction in gastric pressure.

Our patients already reported a higher level of dyspnea (increased MRC) and had lower lung volumes than controls, but with similar BMI and no significant comorbidities that could influence this increased breathlessness. The inspiratory strength was reduced regardless of the side of hemidiaphragm involvement (MIP 59.9% of predicted in RP and 63.4% of predicted in LP), which was confirmed by the other volitional tests. A previous study [4] of UDP found similar values for MIP, around 60% of predicted. Lisboa et al. [20] reported that MIP values are below normal in these patients. Of note, Laroche et al. [4] also considered UDP to be the cause of exercise intolerance and dyspnea, because their patients had no relevant clinical comorbidity that would cause these symptoms.

It is well established that lung volume influences the inspiratory strength and diaphragmatic paralysis is related to a restrictive pattern, which is characterized by reduced FVC [2]. However, correcting the Pdi during bilateral twitch by FVC, patients with UDP persisted with reduced values (2.5 ± 1.7 vs 4.6 ± 2.2 cmH_2_O / L, *p* = 0.001) reinforcing that the low Pdi values are related mainly to diaphragm weakness and not due the lower lung volume. The normalization of Pdi during bilateral twitch by FRC would be also another option, however the FRC was not measured in control group.

Magnetic phrenic nerve stimulation has been described as a useful tool to evaluate diaphragmatic dysfunction [14, 15, 21–23]. However, just one study evaluated the two hemidiaphragms separately, involving 11 patients with recent UDP (symptoms started within 12 months of a normal chest radiograph), and no controls were available to use for comparison [4]. In contrast to our results, the Pdi_Tw_ was reduced in the paralyzed hemidiaphragm but preserved in the non-paralyzed hemidiaphragm. It is important to highlight that some patients with UDP have the possibility of improving the phrenic function in a short period [1, 24]. On the other hand, after 12 months the chance of recovery decreases. Because over half of our patients were evaluated more than 12 months after onset or radiograph alteration, we ascribe the difference between the results to the shorter time of disease in the patients evaluated by Laroche et al. [4]. Probably, the shorter time of disease resulted in a more preserved hemidiaphragm, because even 2 patients had normal phrenic nerve conduction times [4]. Thus, the paralyzed hemidiaphragm possibly was not so flaccid and, as a result, contributed to the greater pressures in the non-paralyzed hemidiaphragm.

Our most important finding was that Pga is the main factor that influences the decrease in Pdi_TW_ in UDP, and it also affects the generation of pressure by the non-paralyzed hemidiaphragm. Mills et al. [14] evaluated that the pattern of pressure changes during Twitch and also found a decline in Pga, justifying that it was due to the diaphragm being sucked up into the chest on inspiration, thus reducing abdominal pressure. However, this study involved only 5 patients who had bilateral diaphragmatic paralysis.

Gibson [25] reported that each hemidiaphragm could apparently operate independently, because “the tension on one side was not well transmitted to the other.” Our USG evaluation confirmed the normal mobility and thickness of the non-paralyzed hemidiaphragm during normal and deep breathing, in which we cannot rule out some influence of accessory inspiratory muscle. However, this independence and the preservation of the non-paralyzed hemidiaphragm were not confirmed by our results during isolated phrenic magnetic stimulation. We believe that Pdi_TW_ and mainly the Pga of the non-paralyzed hemidiaphragm were reduced because the paralyzed hemidiaphragm was not secured and was sucked up into the chest, as described by Mills et al. [14]. Consequently, the non-paralyzed hemidiaphragm could not generate positive pressure in the abdominal compartment and even the pleural pressure was less negative. However, studies are required to confirm this assumption. Because the 2 diaphragmatic crura are joined by a fibrous median arcuate ligament [2], we cannot rule out possible interference of the paralyzed hemidiaphragm decreasing the tension and, therefore, impairing the generation of pressure by the non-paralyzed hemidiaphragm during Twitch stimulation. Finally, adjusting the Pdi to the respective diaphragm atrophy (Thickness_TLC_), we found an inspiratory weakness even more evident in UDP patients.

Bellemare et al. [21] reported that bilateral Pdi is greater than the sum between right plus left Pdi during unilateral stimulus in 6 healthy individuals, but stimulation by the phrenic nerve was electrical and with a different frequency (5-35 Hz). Our results showed similar Pdi during bilateral Twitch compared with the sum of the right and left stimulus, and it was consistently found in all healthy controls. It is probable that the high-frequency phrenic stimulation impacted the results preventing high values in bilateral Pdi. Finally, even in this study [21], the authors suggested there is an upward displacement of the abdominal contents on the normal hemidiaphragm, stretching it.

The diaphragm plication effects reinforce the importance of having a stable hemidiaphragm to allow positive pressure in the abdominal compartment. Surgery plication has the goal of avoiding the dysfunctional movement of the hemidiaphragm during inspiration, with no interference in the force generation by the paralyzed hemidiaphragm. Despite this, studies of diaphragm plication have shown an increase from 10 to 30% in lung function, reduction in dyspnea [26], and an increase in maximal transdiaphragmatic pressure (Pdi_Máx_) [27].

There are relevant findings of respiratory mechanics in diaphragm paralysis induced in dogs [28, 29]. De Troyer et al. [28] using radiographic evaluation found that the electrical stimulation of preserved hemidiaphragm reduces the caudal displacement of the central portion of the preserved contralateral hemidiaphragm. Scillia et al. [29] found a caudal displacement of the paralyzed hemidiaphragm during electrical stimulation of the non-paralyzed hemidiaphragm. However, this caudal movement was observed in dogs with induced injury in the parasternal intercostal muscles besides a pneumothorax, factors that interfere significantly with pressure generation in the pleural space. In this way, there is no negative pressure secondary to the inspiratory accessory muscle recruitment, which in fact sucks the paralyzed hemidiaphragm in a cranial direction.

This study has some limitations. First, the patients had predominantly left hemidiaphragm paralysis, which was found in another study as well [4]. Nevertheless, we have not found any difference in pressures or USG variables between left and right hemidiaphragm involvement. Second, we postulate that the reduced inspiratory strength by the normal hemidiaphragm is related to the cranial movement of the involved muscle; however, this was not confirmed by imaging techniques, and future studies are required to confirm this assumption. Third, although the coils were positioned to create a field more focused on the phrenic nerve, we cannot confirm that the accessory and rib cage muscles were not activated during the stimuli. However, cervical magnetic stimulation would be responsible merely for stiffening the rib cage, and the diaphragm would be allowed to contract against a stable rib cage, acting efficiently [14]. Finally, magnetic phrenic nerve stimulation is considered a useful tool to evaluate more specifically diaphragmatic dysfunction for many studies [14, 15, 21–23].

## Conclusion

The results of the current study show that in symptomatic patients with UDP global inspiratory strength is reduced not only due to the weakness in the paralyzed hemidiaphragm but also to the impairment in the generation of pressure by the non-paralyzed hemidiaphragm. This comes from the flaccidness of the affected side, which does preclude the generation of positive pressure in the abdominal compartment during diaphragm contraction. Studies of diaphragm plication over these physiological mechanisms are highly relevant for gaining a better understanding of the process.

## Abbreviations

BMI: Body mass index
CG: Control group
DL_CO_: Carbon monoxide diffusion capacity
FEV1: Expiratory forced volume in the first second
FRC: Functional residual capacity
FVC: Forced vital capacity
LP: Left paralysis
MEP: Maximal expiratory pressure
MIP: Maximal inspiratory pressure
Pdi: Transdiaphragmatic pressure
Pes: Esophageal pressure
Pga: Gastric pressure
Pmo: Mouth pressure
RP: Right paralysis
RV: Residual volume
TLC: Total lung capacity
Twitch: Phrenic nerve stimulation
UDP: Unilateral diaphragm paralysis
USG: Ultrasound
